# Obesity and Risk of High-Grade Dysplasia and Gallbladder Cancer Among Women with Gallstones in the Chile Biliary Longitudinal Study

**DOI:** 10.3390/cancers18203356

**Published:** 2026-10-16

**Authors:** Paz Cook, Ruth M. Pfeiffer, Jennifer S. Smith, Juan Carlos Araya, Noldy Mardones, Laura Huidobro, Hector Losada, Allan Hildesheim, Juan Carlos Roa, Claudia Marco, Ian Reyes, Matias Pozo, Catterina Ferreccio, Jill Koshiol, the Chile BiLS Study Group

**Affiliations:** 1 Gillings School of Global Public Health, University of North Carolina at Chapel Hill, Chapel Hill, NC 27599, USA; 2 Escuela de Salud Pública, Pontificia Universidad Católica de Chile, Santiago 8330077, Chile; 3 Center for Cancer Prevention and Control (CECAN), Santiago 8331150, Chile; 4 Infections and Immunoepidemiology Branch, Division of Cancer Epidemiology and Genetics, National Cancer Institute, Bethesda, MD 20892, USA; 5 Hospital Dr. Hernán Henríquez Aravena, Temuco 4781151, Chile; 6 Facultad de Medicina, Universidad de La Frontera, Temuco 4781218, Chile; 7 Facultad de Medicina, Universidad Católica del Maule, Talca 3480112, Chile; 8 Independent Researcher, Willis, VA 24380, USA; 9 Facultad de Medicina, Pontificia Universidad Católica de Chile, Santiago 8330077, Chile; 10 Instituto de Salud Pública, Santiago 7780050, Chile

**Keywords:** gallbladder cancer, gallstones, obesity, waist-to-height ratio, central adiposity, Cox proportional hazards models

## Abstract

Background/Objectives: Gallbladder cancer (GBC) remains a leading cause of cancer death in Chilean women. Gallstones are present in >90% of GBC cases, but few individuals with gallstones develop cancer, suggesting additional factors are needed. Methods: To determine whether obesity increases the risk of progression from gallstones to high-grade dysplasia (HGD) and GBC, we used data from the Chile Biliary Longitudinal Study, 2016–2025, comprising women with ultrasound-detected gallstones in high-incidence regions of Chile.We measured body mass index (BMI) and waist-to-height ratio (WHtR) at baseline and up to two follow-up visits. We used Cox proportional-hazards models with age as the time scale to quantify associations of time-varying BMI and WHtR with HGD/GBC risk. Results: During a median follow-up of 4.8 years, 116 of 4711 women developed HGD/GBC. Time-varying BMI was not significantly associated with HGD/GBC risk (*p*-overall = 0.11; *p*-nonlinearity = 0.20). In contrast, time-varying WHtR showed a non-linear association with HGD/GBC (*p*-overall = 0.02; *p*-nonlinearity = 0.02). Baseline WHtR and BMI models showed similar patterns of associations. Based on longitudinal WHtR trajectories among cases and lag sensitivity analyses, preclinical disease-related weight loss is unlikely to fully explain the observed associations. Conclusions: In conclusion, central obesity measured by WHtR was non-linearly associated with HGD/GBC risk in this population, suggesting that central adiposity may impact the progression from gallstones to HGD/GBC.

## Simple Summary

Gallbladder cancer is an important cause of cancer death among women in Chile. Although gallstones are the main risk factor, only a small proportion of women with gallstones develop precancerous lesions or gallbladder cancer. We followed Chilean women with gallstones to investigate whether overall obesity and abdominal obesity were associated with progression to high-grade dysplasia or gallbladder cancer. Overall obesity, measured by body mass index, was not clearly associated with risk. In contrast, abdominal obesity, measured by waist-to-height ratio, showed a non-linear association with high-grade dysplasia or gallbladder cancer. These findings suggest that body fat distribution may provide information beyond overall body size when evaluating progression among women with gallstones. Further studies in other high-risk populations are needed to confirm these findings and determine their potential value for risk assessment.

## 1. Introduction

Gallbladder cancer (GBC) is one of the most lethal malignancies of the gastrointestinal tract, with marked geographic variability [1]. Although rare worldwide, GBC remains a leading cause of cancer deaths in Chile, particularly among women. In 2022, Chilean women had the third-highest estimated global incidence and mortality rates (5.7 and 3.6 per 100,000 population, respectively) [2]. In the regions with high risk (Los Ríos and Los Lagos), standardized incidence rates reach 24.3 per 100,000 in women, exceeding 119 per 100,000 in women ≥ 70 years [3]. 

Gallstones have long been recognized as the predominant risk factor of GBC, present in more than 90 percent of cases [4,5,6]. Gallstone-related GBC typically develops through a sequence of histopathological changes, from high-grade dysplasia (HGD; also termed high-grade biliary intraepithelial neoplasia and historically referred to as carcinoma in situ) and eventually invasive carcinoma [7]. Obesity, measured by body mass index (BMI), has been consistently identified as a metabolic risk factor for gallstones [8], increasing risk by 60–200 percent, especially in women [9,10], and might increase GBC risk directly, for example, by inducing chronic adipose-induced inflammation [11].

Historically, most studies investigating the association between obesity and cancer risk have relied on a single measurement of BMI to characterize obesity. However, accumulating evidence suggests that central adiposity, typically assessed by waist-to-height ratio (WHtR) or waist circumference, may be a more informative marker of cancer risk. Unlike BMI, which primarily reflects overall body size relative to height, WHtR captures abdominal fat distribution and may therefore better reflect visceral adiposity and related metabolic and inflammatory alterations potentially relevant to carcinogenesis [12,13]. Although a meta-analysis has shown that GBC risk increases by 68% in obese women [14], most of the included studies were conducted in general populations. Thus, it remains unclear whether obesity (1) only increases GBC risk by promoting gallstone formation or also increases risk of progression from gallstones to HGD/GBC, and (2) whether overall adiposity (BMI) or central adiposity (e.g., WHtR) is more important.

To clarify whether central and overall obesity act as an independent carcinogenic cofactor for progression to HGD/GBC, beyond their impact on gallstone formation, we leveraged data from the prospective Chile Biliary Longitudinal Study (BILS). Understanding whether obesity acts as an independent cofactor in the progression to HGD/GBC among patients with gallstones is key to developing effective risk-stratification and prevention strategies in this group.

## 2. Methods

### 2.1. Study Design and Population

**Figure 1:**
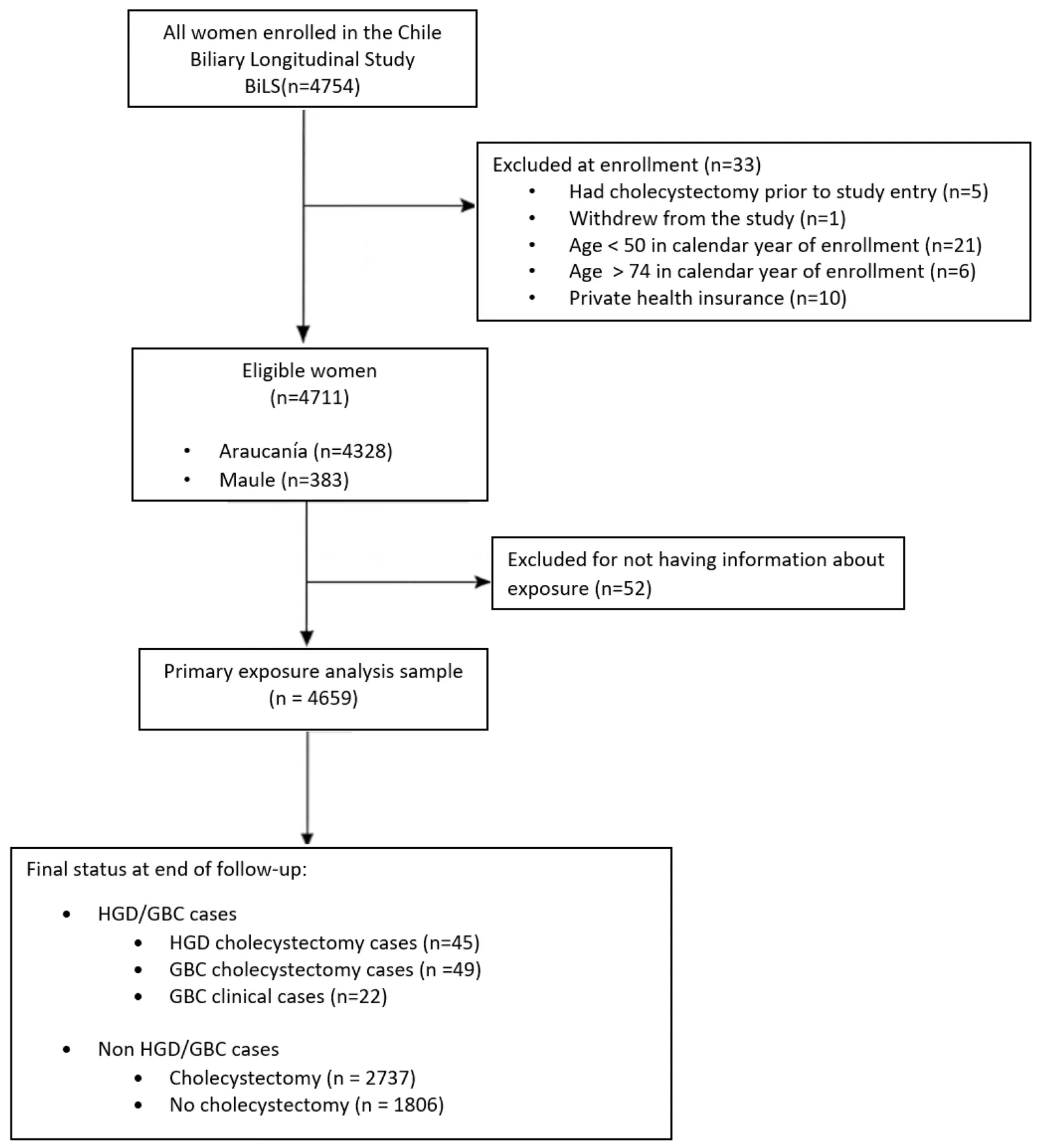
Chile BiLS Flowchart. Figure 1 showing a flowchart of participant selection for the Chile Biliary Longitudinal Study (BiLS). The diagram begins with a box showing all women enrolled in BiLS (*n* = 4754). An arrow leads to a box listing exclusions at enrollment (*n* = 43), including prior cholecystectomy before study entry (*n* = 5), withdrawal from the study (*n* = 1), age under 50 years at enrollment (*n* = 21), age 74 years or older at enrollment (*n* = 6), and private health insurance (*n* = 10). The next box shows the eligible women (*n* = 4711), with two bullets indicating recruitment sites: Araucanía (*n* = 4328) and Maule (*n* = 383). An arrow leads to another box showing women excluded for not having information about exposure (*n* = 52), followed by the primary exposure analysis sample (*n* = 4659). The bottom box summarizes final status at end of follow-up, separating women with and without high-grade dysplasia or gallbladder cancer (HGD/GBC). Among HGD/GBC cases, bullets list HGD cholecystectomy cases (*n* = 45), GBC cholecystectomy cases (*n* = 49), and GBC clinical cases (*n* = 22). Among women without HGD/GBC, bullets list cholecystectomy (*n* = 2737) and no cholecystectomy (*n* = 1806).

Chile BiLS is a prospective cohort of women aged 50–74 years with gallstones, conducted in the Araucanía province and Maule region [15], two areas of Chile with high GBC incidence. Between January 2016 and January 2019, 19,239 women underwent abdominal ultrasound screening performed using Siemens ACUSON P500 and P300 portable ultrasound systems (Siemens Healthineers, Erlangen, Germany). Eligibility required enrollment in the public health system and ultrasound-detected gallstones. Women with a prior cholecystectomy (surgical removal of the gallbladder), a known previous GBC diagnosis, or inability to complete questionnaires were excluded. Recruitment included population-based census visits (*n* = 1092), community campaigns (*n* = 2925), and invitations through local health-system hepatobiliary ultrasound, surgical consultation, and cholecystectomy waiting lists (*n* = 694), totaling 4711 women (Figure 1). Baseline, year 2, and year 4 visits included hepatobiliary ultrasound, anthropometry, blood pressure, blood collection, and questionnaires. 

The eligible cohort included 4711 women, whose baseline characteristics are presented in Table 1. After excluding 52 participants (1.1%) without baseline obesity measures, 4659 women were eligible for the primary exposure analyses (Figure 1). The four-year visit response rate was 94%. This project was approved by institutional review boards from all institutions involved. All participants provided written informed consent. 

Follow-up extended from age at cohort entry to age at cholecystectomy, death, HGD/GBC diagnosis, loss to follow-up, or study end (August 2025), whichever occurred first. After completion of the scheduled baseline, 2-year, and 4-year visits, hospital surgery lists from the Araucanía province’s hospitals were reviewed daily on weekdays to identify scheduled cholecystectomies. When surgery was scheduled, the field staff conducted a cholecystectomy visit at or shortly before the time of surgery. Incident GBC cases and deaths were also identified (see details below). 

### 2.2. Exposure Assessment

Anthropometric measures were collected at baseline, 2- and 4-year visits by trained staff using standardized protocols. Weight, waist circumference, hip circumference, and height were measured. We studied (1) BMI, computed as weight (kg)/height (m)^2^, and (2) central obesity, assessed by WHtR, computed as waist circumference/height (cm) as the main exposure variables. 

We evaluated BMI using three approaches. First, BMI (kg/m^2^) was categorized using standard World Health Organization (WHO) categories (Underweight/normal (combined), Overweight, Obesity classes I, II, and III). Second, BMI was evaluated as a continuous variable using restricted cubic splines, with knots at 28.1, 31.7, 35.6, and 40.0, corresponding to the 25th, 50th, 75th and 90th percentiles. Third, in an exploratory analysis, cut-points were selected based on visual inspection of changes in the shape and direction of the fitted continuous restricted cubic spline curve, and BMI was evaluated categorically using five “BMI Chile BiLS” categories (15.9–28.0; 28.1–34.0; 34.1–36.0; 36.1–40.0; 40.1–71.2).

We evaluated WHtR using four approaches. First, WHtR was categorized into quartiles (Table 1). Second, WHtR was dichotomized using the conventional cutoff of 0.50 (≤0.50 vs. >0.50). Third, WHtR was evaluated as a continuous variable using restricted cubic splines. Knots were placed at the 25th, 50th, 75th, and 90th percentiles (0.60, 0.65, 0.71, and 0.77). Fourth, in an exploratory analysis, cut-points were selected based on visual inspection of changes in the shape and direction of the fitted continuous restricted cubic spline curve, and WHtR was evaluated categorically using five “WHtR Chile BiLS” categories (0.38–0.60; 0.61–0.68; 0.69–0.72; 0.73–0.77; 0.78–1.22). These spline-informed categories were used descriptively to facilitate interpretation of the continuous exposure–response patterns and were not intended to represent validated clinical or population-specific cut-points.

For both BMI and WHtR, the overall association was evaluated using a Wald test for the restricted cubic spline terms, whereas departure from linearity was evaluated using a likelihood ratio test comparing the restricted cubic spline model with the corresponding linear model.

### 2.3. Outcome Ascertainment

The primary outcome was the composite of HGD/GBC. Cases were classified as (1) cholecystectomy HGD/GBC cases if diagnosed through pathology review of gallbladder tissue obtained at cholecystectomy (81.0%) or (2) clinically detected GBC cases (19.0%), defined as invasive GBC diagnosed in the absence of a cholecystectomy-based pathological diagnosis. For clinically detected GBC cases, a comprehensive review of each participant’s medical record was performed, and relevant diagnostic information was abstracted, including computed tomography (CT) and magnetic resonance imaging (MRI) findings, oncology committee assessments, and death certificate information, when available. The collected clinical documentation was systematically reviewed by a BiLS expert panel, which evaluated the available evidence to determine final case eligibility and classification. Outcome ascertainment involved active surveillance throughout follow-up, including regular review of surgical lists from participating public and private hospitals in the study region, attendance by study personnel at surgeries to facilitate collection of gallbladder tissue specimens, review of clinical records, and linkage with the national death registry. Participants could also contact the study team when they were scheduled for surgery. For HGD/GBC detected through cholecystectomy, diagnoses were based on histopathologic evaluation of the surgical specimen; 87.5% of these cases additionally underwent complete mapping of the gallbladder tissue.

### 2.4. Statistical Analysis

Potential confounders were identified a priori through a review of the epidemiologic literature conducted during study planning, in which candidate variables were evaluated according to prior evidence of their relationships with adiposity and HGD/GBC and considered within a conceptual causal framework.

A priori candidate set included parity (0, 1, …, 14); FONASA national health-insurance group (classified as: A = indigent/non-contributors, public-only; B = low-income contributors; C = middle-income; D = highest copays in the public system [not necessarily wealthy]); first-degree family history of gallbladder cancer (0, 1, or ≥2 relatives); educational attainment (≤8, 9–12, or ≥13 years); number of lost teeth (0, 1–6, 7–13, 14–24, or >24); smoking (never, former, or current); alcohol use (never, former, or current); fried food consumption (0, 1, or ≥2 times/week); and self-reported Mapuche Amerindian ethnicity, regular NSAID use including aspirin, current statin use, heart disease, diabetes, fatty liver disease, physical activity in the last month, lifetime physical activity, hysterectomy, use of contraceptives, regular corticosteroid use, regular antibiotic use, and use of pesticides (each yes/no).

Associations of potential confounders with HGD/GBC were evaluated using Cox proportional-hazards models with age as the time scale. Associations with obesity were evaluated using polytomous logistic regression for BMI categories and age-adjusted linear regression for continuous WHtR. Within this a priori-defined candidate set, covariates associated with either BMI or WHtR and with HGD/GBC at *p* ≤ 0.10 were considered for multivariable Cox modeling. Backward selection using a bias–precision trade-off approach [16] was used to determine the final parsimonious adjustment set given the limited number of HGD/GBC outcomes. Parity and Mapuche Amerindian ethnicity were retained for both BMI and WHtR. FONASA was additionally retained a priori because of its relevance to socioeconomic position and health care access and utilization.

We fit multivariable Cox proportional-hazards models using age as the time scale to estimate adjusted hazard ratios for the association between obesity measures and HGD/GBC (aHRs, 95% confidence intervals, CIs, PROC PHREG, SAS 9.4). Participants started follow-up at their age at enrollment and exited at the age of HGD/GBC diagnosis or censoring (age of cholecystectomy, death, loss to follow-up, or study end). We assessed associations for time-varying BMI and WHtR (updated at the 2- and 4-year visits). For participants who underwent cholecystectomy before visit 2 (30%) or visit 4 (46%), obesity measurements were taken from their last visit before censoring. Cases with missing covariate data (*n* = 5, 0.3%) were excluded from the analysis. The proportional-hazards assumption for fixed covariates was evaluated using covariate-time interactions and visual inspection of log(–log S) versus log(time) plots. No substantial violations of the proportional hazards assumption were identified. To assess potential reverse causation due to undiagnosed or preclinical disease present near cohort entry, we conducted sensitivity analyses using the spline-informed WHtR categories, excluding HGD/GBC events occurring within the first 6 months, 1 year, and 2 years of follow-up. 

To evaluate longitudinal trends in central obesity, we constructed trajectories of WHtR for women progressing to a HGD/GBC diagnosis or cholecystectomy. WHtR values at baseline were grouped into the five Chile BiLS categories described above, and repeated measures of WHtR were analyzed over time anchored at the date of diagnosis or surgery (year 0). Mixed-effects linear regression models with participant-specific random intercepts were used to estimate the annual rate of change (slope) in WHtR within each baseline category using PROC MIXED, SAS 9.4. Mean predicted trajectories with 95% CIs and fitted linear trends were plotted for each of the three highest WHtR categories to facilitate comparison of changes in central obesity preceding a GBC/HGD diagnosis or cholecystectomy using PROC SGPANEL, SAS 9.4. To assess the robustness of our primary findings, we conducted outcome-specific analyses for HGD, cholecystectomy-detected GBC, and clinically detected GBC; an analysis restricted to cholecystectomy-detected HGD/GBC outcomes, for which outcome status was determined histopathologically; and case–case analyses evaluating potential differences by outcome type and mode of ascertainment. Case–case analyses were performed using multivariable logistic regression and were adjusted for age at enrollment, parity, FONASA health coverage, and self-reported Mapuche Amerindian ancestry. Results of these additional analyses are presented in the Supplementary Materials.

## 3. Results

### Baseline Characteristics

Among the 4711 eligible women in the Chile BiLS cohort, most had completed 8 or fewer years of schooling (Table 1). Nearly all women were in the overweight or obese BMI categories (median BMI 31.4 kg/m^2^). WHtR values indicated widespread central obesity (median WHtR 0.65). During follow-up, 4595 participants remained free of HGD/GBC and 116 developed GBC or HGD, including 94 HGD/GBC cases identified through cholecystectomy and 22 clinical GBC cases. Women who developed HGD/GBC were slightly older at baseline than non-cases. Mapuche Amerindian ethnicity was more frequent among women who developed HGD/GBC than among non-cases, with the highest proportion observed among clinical GBC cases. Low education and being insured with FONASA group A (health coverage, representing the lowest income group) were more common in HGD/GBC cases (Table 1). 

Among the 4659 women eligible for the primary exposure analyses, the median follow-up was 4.78 years (range, 0.01–10.17), with a total accumulated follow-up time of 22,296.2 person-years. The median BMI was 31.0 kg/m^2^ in cholecystectomized non-cases (*n* = 2763) and 32.9 kg/m^2^ in non-cholecystectomized non-cases (*n* = 1832) compared to 31.0 kg/m^2^ in cholecystectomized cases (*n* = 94) and 30.9 kg/m^2^ in clinical cases (*n* = 22). Central adiposity was slightly higher in cases, with a median WHtR of 0.66 in cholecystectomized cases and 0.67 in clinical cases versus 0.64 and 0.66 in cholecystectomized and non-cholecystectomized non-cases, respectively. High parity was more common among cases than non-cases (Table 1).

In multivariable analysis adjusted for parity, FONASA health coverage, Mapuche ethnicity, when modeled linearly, continuous BMI was not statistically significantly associated with risk of HGD/GBC (aHR = 0.97, 95% CI: 0.94–1.00). Similarly, when modeled as a single linear term, continuous WHtR was not associated with increased risk of HGD/GBC (aHR = 0.21, 95% CI: 0.02–1.92). Using BMI categorized using WHO criteria with class I obesity (BMI 30.0–34.9 kg/m^2^) as the reference group, the other BMI categories did not show statistically significant associations with HGD/GBC risk (global *p* = 0.41) (see Table 2, Panel A). Removing underweight individuals (*n* = 6) from the analysis did not impact the results.

**Figure 2:**
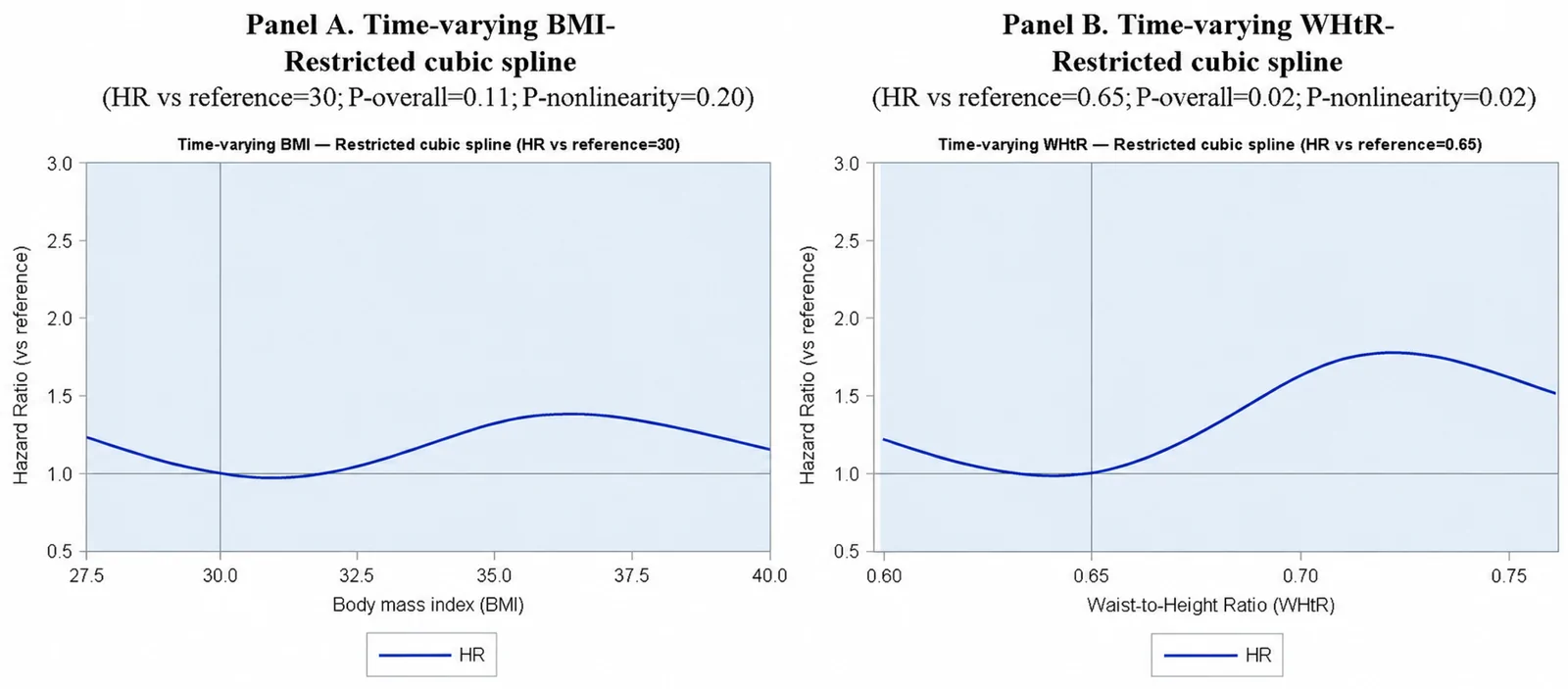
Time-varying body mass index and waist-to-height ratio in relation to risk of gallbladder cancer and high-grade dysplasia (HGD/GBC) in Chilean women (2016–2025), modeled with restricted cubic spline Cox regression (Panel (**A**): BMI (*n* = 4517); Panel (**B**): WHtR (n = 4523)). Figure 2 showing time-varying body mass index (BMI, *n* = 4517) and waist-to-height ratio (WHtR, *n* = 4523) in relation to risk of gallbladder cancer or high-grade dysplasia in Chilean women (2016–2025), modeled using restricted cubic spline Cox regression. The figure contains two side-by-side panels: Panel (**A**) for BMI and Panel (**B**) for WHtR. In Panel A, the *x*-axis shows BMI values in kg/m^2^ and the *y*-axis shows the hazard ratio for HGD/GBC, with a blue smooth spline curve representing the estimated hazard ratio across BMI values relative to a reference BMI of 30; vertical reference lines highlight the chosen reference point. In Panel (**B**), the *x*-axis shows WHtR values and the *y*-axis shows the hazard ratio for HGD/GBC, with a similar blue smooth spline curve representing the estimated hazard ratio relative to a reference WHtR of 0.65. Footnote: *p*-overall values correspond to Wald tests for the overall association of time-varying BMI (Panel A, *p*-overall = 0.11) and WHtR (Panel B, *p*-overall = 0.02) with HGD/GBC risk. *p*-nonlinearity values were obtained from likelihood ratio tests comparing the restricted cubic spline and linear models (BMI, *p*-nonlinearity = 0.20; WHtR, *p*-nonlinearity = 0.02).

Next, we fit a multivariable Cox proportional hazards model with continuous, time-varying BMI modeled using restricted cubic splines. BMI was not significantly associated with HGD/GBC risk (*p*-overall = 0.11), with no evidence of departure from linearity (*p*-nonlinearity = 0.20) (Figure 2A). In an exploratory analysis, we used the observed spline shape to define five BMI categories, with women in the second category (BMI 28.1–34.0 kg/m^2^) as the reference group (Table 2, Panel B). These spline-informed categories showed no overall association with HGD/GBC risk (*p*-overall = 0.36). Compared with women with BMI 28.1–34.0 kg/m^2^, HRs were 1.45 (95% CI, 0.92–2.31) for BMI 15.9–28.0 kg/m^2^, 1.12 (95% CI, 0.59–2.14) for BMI 34.1–36.0 kg/m^2^, 1.23 (95% CI, 0.70–2.17) for BMI 36.1–40.0 kg/m^2^, and 0.74 (95% CI, 0.36–1.53) for BMI 40.1–71.2 kg/m^2^.

To evaluate central adiposity, we first modeled WHtR using quartiles (Table 3, Panel A), with Q2 (0.60–0.65) as the reference category, and adjusted for parity, health coverage groups, and Mapuche Amerindian ethnicity. Across quartiles, there was no clear trend in HGD/GBC risk (global *p* = 0.11). Women in Q1 (0.38–0.59; HR 1.74, 95% CI: 0.99–3.06) and Q4 (0.72–1.22; HR 1.48, 95% CI: 0.88–2.57) had somewhat higher, but imprecisely estimated, risks compared with Q2, while Q3 (0.66–0.71) had similar risk as women in Q2 (HR 1.00, 95% CI: 0.54–1.83). Similarly, WHtR dichotomized at the conventional cutoff of 0.50 was not associated with HGD/GBC risk (>0.50 vs. ≤0.50: aHR = 0.70; 95% CI, 0.22–2.21; *p* = 0.54).

Next, we fit a Cox proportional hazards model with continuous, time-varying WHtR modeled using restricted cubic splines. WHtR was significantly associated with HGD/GBC risk (*p*-overall = 0.02), with evidence of departure from linearity (*p*-nonlinearity = 0.02) (Figure 2B). In an exploratory analysis, we used the observed spline shape to define five WHtR categories (Table 3, Panel B). Compared with women in the reference category (0.61–0.68), women in the lowest WHtR category (0.38–0.60) had a higher risk of HGD/GBC (aHR = 1.79; 95% CI, 1.08–2.97). The aHR was also elevated for WHtR 0.73–0.77 (aHR = 1.73; 95% CI, 0.96–3.12), although the confidence interval included the null. The overall association across the spline-informed WHtR categories was not statistically significant (*p*-overall = 0.10).

Global *p*-values correspond to Type 3 Wald tests, two-sided. To assess potential reverse causation from preclinical disease, we repeated the analyses using the spline-informed WHtR categories, after excluding HGD/GBC events occurring within the first 6 months, 1 year, and 2 years of follow-up. The pattern and magnitude of the WHtR associations were generally similar across lag periods, although confidence intervals widened as the number of events decreased (101, 93, and 73 events, respectively). Increasing the lag period did not result in progressive attenuation of the associations (Table S1).

Anchoring at time of HGD/GBC diagnosis (time 0), WHtR trajectories among cases (*n* = 116; 235 visits) showed a small increase in WHtR as the time to HGD/GBC diagnosis approached (*p* = 0.05). Estimated slopes were positive across Chile BiLS categories, but differences in slopes by WHtR categories were not statistically significant. High WHtR categories (Cat3 and Cat4) remained consistently high throughout follow-up (Figure S1).

Among 2828 participants contributing 5333 WHtR observations anchored at the date of cholecystectomy (time 0), trajectories differed by Chile BiLS categories of WHtR (*p* < 0.001). In lower categories (Cat1 and Cat 2) of BiLS, WHtR rose as surgery approached. For the middle Chile BiLS WHtR category the trajectory was flat, while the slope was negative in higher categories, indicating that WHtR tended to decline over time for women with the highest WHtR (Figure S2). In the highest baseline WHtR category (Cat 5), the estimated slope was −0.00181 units/year (95% CI, −0.00348 to −0.00014), corresponding to an estimated absolute change of approximately −0.0075 WHtR units over the median 4.13 years from enrollment to cholecystectomy (Figure S2).

Outcome-specific analyses for HGD, cholecystectomy-detected GBC, and clinically detected GBC yielded imprecise estimates because of the limited number of events within each outcome type. Across spline-informed BMI categories, associations were more apparent for cholecystectomy-detected GBC (Table S2). For WHtR categories, associations were more apparent for HGD and cholecystectomy-detected GBC than for clinically detected GBC, although outcome-specific estimates were imprecise (Table S3). When HGD and GBC detected at cholecystectomy were combined, associations across WHtR categories were generally consistent with the primary findings (Table S3).

In case–case analyses restricted to cholecystectomy-detected outcomes, there was no evidence of an overall difference between HGD and surgically detected GBC according to baseline WHtR category (*p*-overall = 0.23) (Table S4). Similarly, in case–case analyses comparing HGD/surgically detected GBC with clinically detected GBC, there was no evidence of an overall association between mode of outcome ascertainment and baseline WHtR category (*p*-overall = 0.99) (Table S5).

## 4. Discussion

To our knowledge, this is the first prospective cohort to assess the association of both overall and central obesity with HGD/GBC risk in a high-risk population. Among nearly 5000 women with gallstones in the Chile BiLS cohort, we identified 116 women with gallbladder cancer or high-grade dysplasia (HGD/GBC) over a median follow-up time of 4.8 years. Over half of participants were clinically obese (BMI ≥ 30.0 kg/m^2^) with relatively high overall and central obesity (waist-to-height ratios, WHtR) measurements at baseline. HGD/GBC risk was not related to differing levels of BMI or quartiles of WHtR. However, a non-linear association was observed between central obesity and HGD/GBC risk when using the continuous restricted cubic spline analysis (*p*-overall = 0.02; *p*-nonlinearity = 0.02). The fitted spline displayed an S-shaped exposure–response pattern. In contrast, time-varying BMI was not significantly associated with HGD/GBC risk (*p*-overall = 0.11), with no evidence of departure from linearity (*p*-nonlinearity = 0.20). Outcome-specific analyses suggested broadly similar patterns for HGD and cholecystectomy-detected GBC, although estimates were imprecise because of the limited number of events within each outcome type. Estimates for clinically detected GBC were particularly imprecise.

Unlike previous studies [14,17,18], we did not see consistent associations between BMI categories and increased risk of GBC, suggesting that much of the previously observed risk could be driven by increased risk of gallstones due to obesity. WHtR, however, demonstrated a more subtle association among women with gallstones, suggesting that central adiposity may impact HGD/GBC development among gallstone carriers.

Overall, the risk of HGD/GBC followed a non-linear pattern across different levels of central obesity. The higher risk among less obese women may be explained by these women undergoing cholecystectomy earlier in the study. Less obese women were more likely to be considered better surgical candidates because of lower cardiovascular and perioperative risk, which may increase incidental detection of HGD/GBC. In contrast, women in the highest WHtR category had a lower HGD/GBC risk, which may be explained by the lower rate of cholecystectomies in this group, resulting in lower detection rates. It may also be influenced by women in this category being more likely to exit follow-up because of death from causes other than HGD/GBC, which is well described in the literature [19,20]. We did not directly evaluate these mechanisms, and therefore this explanation remains speculative.

In addition, women with very high baseline WHtR who underwent cholecystectomy experienced a modest decline in WHtR prior to surgery. The reason for this modest decline cannot be determined from these observational data and should not be interpreted as evidence of a causal effect of surgical eligibility or preclinical disease. Furthermore, WHtR trajectories showed no substantial decline preceding HGD/GBC diagnosis, and sensitivity analyses using the spline-informed WHtR categories and excluding events occurring within the first 6 months, 1 year, and 2 years of follow-up did not show progressive attenuation of the association. These findings provide some reassurance that preclinical disease-related weight loss is unlikely to fully explain the observed associations, although reverse causation cannot be completely excluded.

Although previous studies have reported positive associations between higher BMI and GBC [14,21], accumulating evidence suggests that central adiposity measures, particularly waist-to-height ratio, are stronger markers of risk. WHtR captures the distribution of body fat rather than overall body size [13,21], and therefore reflects the visceral and ectopic fat distribution [22,23], which may be a more important driver of inflammatory risk pathways in GB disease [11]. Our findings similarly suggest that central and overall adiposity may have different associations with HGD/GBC risk. The continuous spline analysis, and Chile BiLS categories, identified an association for WHtR but not BMI, although these findings require replication. Similarly, a large multi-center pooling analysis of 27 prospective cohorts including more than 2.7 million adults found that increasing waist circumference, hip circumference, waist-to-hip ratio, and waist-to-height ratio were stronger and more consistently associated with higher risks of gallbladder and intrahepatic bile duct cancers than BMI, for which risk estimates were generally weaker [21]. The proposed direct mechanisms for the effect of central obesity and HGD/GBC include chronic adipose-induced inflammation, hyperinsulinemia, oxidative stress, and upregulation of carcinogenic mediators [11,24]. Experimental evidence indicates that hormonal pathways related to growth factors and sex steroids may promote gallbladder epithelial proliferation [11].

We did not observe an association when using WHtR quartiles or the conventional cutoff of 0.5, likely because almost all participants exceeded this threshold given the very high level of central obesity in our sample (median WHtR 0.65, range 0.38–1.22). In such an extremely obese population, the 0.5 cutoff provides little discrimination, and quartiles may group women with similarly elevated WHtR values, attenuating potential dose–response patterns described in the literature [17]. In addition, given the limited number of clinically detected GBC cases in our cohort, estimates for clinical GBC by WHtR strata were imprecise and should be interpreted with caution. 

Many previous studies have reported a positive association between BMI and GBC [14,17,18,25,26,27]. The lack of association between BMI and HGD/GBC in the current study suggests that previously reported BMI–GBC associations in general populations may be driven largely by the strong link between BMI and gallstone formation [28]. It could also reflect the narrow range of BMI in this population. Central obesity (as captured by WHtR), however, appears to play an additional, more complex role in progression from gallstones to HGD/GBC.

This study has several notable strengths. We systematically evaluated measures of obesity over time. Our study also benefits from robust characterization of exposures and outcomes, as well as a high degree of follow-up completeness, reflected in 94% response rate at the four-year visit [15]. In addition, the amount of missing data in the cohort was less than 3%. Importantly, studying women with established gallstones addresses a clinically and etiologically distinct question—what factors influence subsequent neoplastic progression—analogous to studies of cervical carcinogenesis among women with HPV infection or hepatocellular carcinoma among individuals with chronic HBV or HCV infection [29].

Several limitations should also be considered. First, most of the Chilean women with gallstones were very obese, limiting generalizability to other populations. Because the cohort was restricted to women with gallstones, both the exposure and various risk factors may influence entry into the cohort and subsequent cholecystectomy, which could potentially produce collider bias in which obesity and other risk factors jointly affect both eligibility for the cohort and the likelihood of undergoing cholecystectomy, distorting the observed association between central obesity and HGD/GBC risk. The small number of events also limited statistical power for subgroup and outcome-specific analyses, particularly among clinical GBC cases. Second, limitations related to outcome ascertainment should be considered. Clinically detected GBC cases lacked cholecystectomy-based pathological confirmation; however, comprehensive medical-record review and expert-panel adjudication were used to determine final case eligibility and classification. Nevertheless, some outcome misclassification remains possible. Despite extensive active surveillance and record-based ascertainment, incomplete detection of HGD/GBC cannot be entirely excluded. If the likelihood of outcome detection differed according to adiposity or related factors, the resulting differential ascertainment could have biased the estimated associations; however, the direction and magnitude of this potential bias are uncertain. Similarly, although women with a known previous GBC diagnosis were excluded at enrollment, some participants may have had undiagnosed HGD or preclinical GBC at baseline, particularly because HGD is generally asymptomatic and requires histopathologic examination for detection. Such baseline disease could introduce bias, although its direction and magnitude are uncertain. Third, the duration of follow-up and the role of cholecystectomy warrant consideration. The median follow-up of 4.8 years may have also been insufficient to capture the full progression from gallstones to HGD/GBC in some participants. Women who underwent cholecystectomy without HGD/GBC had a shorter median follow-up (2.59 years) and a lower prevalence of class III obesity (7.1%) than women who did not undergo cholecystectomy (13.9%). If women with very high BMI were less likely to undergo cholecystectomy during follow-up, some HGD could have remained undetected, potentially attenuating associations at the highest levels of adiposity. More broadly, cholecystectomy has a complex role because it both provides the opportunity for histopathologic detection of HGD/GBC and, among women without HGD/GBC, removes the gallbladder and terminates subsequent risk of GBC. Consequently, informative censoring may have influenced our findings if women censored through cholecystectomy differed systematically from those who remained under observation. We did not perform inverse-probability-of-censoring weighting or a competing-risk/multi-state analysis to formally account for this process. In case–case sensitivity analyses, baseline WHtR was not associated overall with mode of outcome ascertainment; however, these analyses were restricted to identified cases and therefore cannot exclude informative censoring in the full cohort. 

Finally, residual confounding remains possible, including from measured factors not retained in the final adjustment set through the statistical selection procedure and from unmeasured or incompletely characterized environmental exposures [30]. 

## 5. Conclusions

In conclusion, central adiposity measured by WHtR was non-linearly associated with HGD/GBC risk among individuals with gallstones. Associations with BMI were less apparent. Our findings highlight the importance of recognizing non-linearity particularly in groups with high obesity prevalence such as Chilean women with gallstones. They also suggest that central, rather than overall, adiposity may be especially informative for characterizing HGD/GBC risk among women with gallstones. Future research directions include replicating these analyses in other high-risk populations to determine whether the observed associations persist across diverse cohorts and ancestries and to evaluate the potential relevance of central adiposity for risk stratification.

## Figures and Tables

**Table 1:** Baseline characteristics of Chile Biliary Longitudinal Study (BiLS) cohort women with gallstones, 2016–2025 (*n* = 4711).

	Overall (*n* = 4711)		Non-Cases (*n* = 4595)				HGD/GBC * Cases (*n* = 116)			
			Cholecystectomy, No HGD/GBC (*n* = 2763)		No Cholecystectomy, No HGD/GBC (*n* = 1832)		Cholecystectomy, HGD/GBC (*n* = 94)		Clinical GBC (*n* = 22)	
Age, years										
Mean (SD)	59.3 (6.6)		58.7 (6.3)		60.1 (6.8)		60.2 (6.2)		62.3 (6.5)	
Median	59		58.0		59.0		61.0		63.5	
Ethnicity ^a^										
No Mapuche Amerindian	3421	72.6	1992	72.1	1353	73.9	66	70.2	10	45.5
Mapuche Amerindian	1185	25.2	710	25.7	437	23.9	27	28.7	11	50.0
Missing or unknown ^b^	105	2.2	61	2.2	42	2.3	1	1.1	1	4.5
Education, no. of years completed										
0–8	2510	53.3	1396	50.5	1047	57.2	51	54.3	16	72.7
9–12	1739	36.9	1080	39.1	619	33.8	36	38.3	4	18.2
≥13	383	8.1	242	8.8	132	7.2	7	7.5	2	9.1
Missing or unknown ^b^	79	1.7	45	1.6	34	1.9	0	0.0	0	0.0
Health coverage ^c^										
FONASA group A	2010	42.7	1124	40.7	827	45.1	48	51.1	11	50.0
FONASA group B	1573	33.4	951	34.4	589	32.2	27	28.7	6	27.3
FONASA group C & D	568	12.0	380	10.8	175	9.6	11	11.7	5	22.7
Other/Unknown FONASA	418	8.9	228	8.3	182	9.9	5	5.3	0	0.0
Missing or unknown ^b^	142	3.0	80	2.9	59	3.2	3	3.2	0	0.0
Anthropometric measurements										
Body mass index (kg/m^2^) ^d^										
Mean (SD)	32.1 (5.9)		31.6 (5.4)		32.1 (6.5)		31.7 (6.0)		31.2 (5.3)	
Median	31.4		31.0		32.9		31.0		30.9	
Range	15.9–71.2		15.9–56.0		16.0–71.2		21.5–47.7		23.4–42.3	
BMI WHO categories	No.	%	No.	%	No.	%	No.	%	No.	%
Under/Normal weight (15.9–24.9)	424	9	255	9.2	154	8.4	13	13.8	2	9.1
Overweight (25.0–29.9)	1420	30.1	896	32.4	490	26.8	27	28.7	7	31.8
Obese (≥30.0)										
Class I (30.0–34.9)	1500	31.8	896	32.4	570	31.1	25	26.6	9	40.9
Class II (35.0–39.9)	852	18.1	492	17.8	336	18.3	21	22.3	3	13.6
Class III (40.0–71.2)	459	9.7	196	7.1	254	13.9	8	8.5	1	4.5
Missing or unknown	56	1.2	28	1.0	28	1.5	0	0.0	0	0.0
Waist-to-height ratio (WHtR) ^e^										
Mean (SD)	0.66 (0.09)		0.65 (0.08)		0.67 (0.09)		0.66 (0.09)		0.67 (0.08)	
Median	0.65		0.64		0.66		0.66		0.67	
Range	0.38–1.22		0.38–0.98		0.41–1.22		0.38–0.85		0.52–0.79	
WHtR Quartiles ^f^	No.	%	No.	%	No.	%	No.	%	No.	%
Q1 (WHtR 0.38–0.59)	1163	24.7	753	27.3	378	20.6	27	28.7	5	22.7
Q2 (WHtR 0.60–0.65)	1158	24.6	705	25.5	432	23.6	17	18.1	4	18.2
Q3 (WHtR 0.66–0.71)	1165	24.7	708	25.6	434	23.7	18	19.2	5	22.7
Q4 (WHtR 0.72–1.22)	1173	24.9	571	20.7	562	30.7	32	34.0	8	36.4
Missing or unknown	52	1.1	26	0.9	26	1.4	0	0.0	0	0.0
Parity ^a^										
0	321	6.8	190	6.9	126	6.9	4	4.3	1	4.5
1–2	1646	34.9	1021	37.0	590	32.2	29	30.9	6	27.3
3–4	1922	40.8	1147	41.5	729	39.8	36	38.3	10	45.5
5–14	749	15.9	363	13.1	357	19.5	24	25.5	5	22.7
Missing or unknown ^b^	73	1.6	42	1.5	30	1.6	1	1.1	0	0
Follow-up from January 2016 to August 2025: years (*n* = 4659) ^g^										
Mean (SD)	4.79 (2.98)		3.02 (2.13)		7.56 (1.78)		3.54 (2.12)		2.69 (2.22)	
Median	4.78		2.59		7.96		3.44		2.12	
Range	0.01–10.17		0.01–9.53		0.04–10.17		0.07–9.59		0.11–7.15	

* Abbreviations: GBC, gallbladder cancer; HGD, high-grade dysplasia; WHtR, waist-to-height ratio; BMI, body mass index; FONASA, Fondo Nacional de Salud. Footnote: Table 1 describes the 4711 eligible cohort participants. The number of participants contributing to individual characteristics varies according to data availability, as indicated by the corresponding missing/unknown categories and footnotes. The follow-up summaries are based on the 4659 participants with baseline obesity measures who were eligible for the primary exposure analyses. Non-case participants are further classified according to cholecystectomy status (*n* = 2763 and *n* = 1832, respectively); HGD/GBC cases are classified as cholecystectomy-detected (*n* = 94) or clinically detected GBC (*n* = 22). ^a^ Self-reported via baseline risk factor questionnaire. ^b^ Includes missing data and the response “Participant doesn’t know/doesn’t respond.”. ^c^ FONASA is Chile’s public health insurance system. Groups A–D are hierarchical classifications used to determine the proportion of health-care costs covered by the government, based on taxable individual income. FONASA group A has the lowest income level and receives the highest level of governmental coverage of health-care costs. “Other health coverage” represents governmental health coverage provided specifically for the Chilean armed forces or police service or other insurance. ^d^ Weight (kg)/height (m)^2^. ^e^ Waist (cm)/height (cm). ^f^ Values are expressed in quartiles. ^g^ Follow-up summaries are based on the 4659 participants with baseline obesity measures who were eligible for the primary exposure analyses (those with available information for WHtR and BMI at baseline).

**Table 2:** Adjusted time-dependent Cox models (age as the underlying timescale) for gallbladder cancer and high-grade dysplasia (HGD/GBC) according to body mass index (BMI, Panel A) and Chile BiLS categories (Panel B), Chile Biliary Longitudinal Study (BiLS), 2016–2025 (*n* = 4517 women; 111 events).

Panel A. BMI WHO Categories (Standard Classification)				Panel B. BMI Chile BiLS Categories (Spline-Informed)			
Variable	*n* (%)	HGD/GBC Events (111)	Adjusted HR (95% CI)	Variable	*n* (%)	HGD/GBC Events (111)	Adjusted HR (95% CI)
BMI WHO categories				BMI Chile BiLS categories			
Underweight/normal (15.9–24.9)	418 (9.3)	15 (13.5)	1.72 (0.85–3.47)	15.9–28.0	1161 (25.7)	33 (29.7)	1.45 (0.92–2.31)
Overweight (25.0–29.9)	1368 (30.3)	31 (27.9)	1.57 (0.96–2.57)	28.1–34.0	1834 (40.6)	40 (36.0)	1.00 (ref)
Obesity class I (30.0–34.9)	1447 (32.0)	33 (29.7)	1.00 (ref)	34.1–36.0	482 (10.7)	12 (10.8)	1.12 (0.59–2.14)
Obesity class II (35.0–39.9)	836 (18.5)	23 (20.7)	1.24 (0.70–2.18)	36.1–40.0	594 (13.2)	17 (15.3)	1.23 (0.70–2.17)
Obesity class III (40.0–71.2)	448 (9.9)	9 (8.1)	1.14 (0.60–2.18)	40.1–71.2	446 (9.9)	9 (8.1)	0.74 (0.36–1.53)
Global *p*	0.41			Global *p*	0.36		

Abbreviations: GBC, gallbladder cancer; HGD, high-grade dysplasia; BMI, body mass index; HR, hazard ratio; CI, confidence interval. All the frequencies are from baseline, while the HRs are from a model with time-varying BMI. Footnote: The analytic sample was restricted to participants with available BMI and covariate data (*n* = 4517). Of the 116 HGD/GBC cases identified during follow-up, 111 were included in the adjusted analyses; five cases were excluded because of missing covariate data. Models were estimated using time-dependent Cox proportional hazards regression with age as the timescale. All models were adjusted for parity (continuous), health coverage (FONASA), and self-reported Mapuche. Amerindian ancestry. Panel A uses standard WHO BMI categories. Panel B uses Chile BiLS-specific BMI categories, defined based on the shape of the restricted cubic spline fit for time-varying BMI in the Chile BiLS cohort. Sample size differs slightly from WHtR models due to missing BMI. Global *p*-values correspond to Type 3 Wald tests, two-sided.

**Table 3:** Adjusted time-dependent Cox models (age as the underlying timescale) for gallbladder cancer and high-grade dysplasia (HGD/GBC) according to waist-to-height ratio (WHtR), Chile BiLS, 2016–2025 (*n* = 4523 women; 111 events).

Panel A. WHtR Quartiles (Distribution-Based Comparison)				Panel B. WHtR Chile BiLS Categories (Spline-Informed)			
Variable	*n* (%)	HGD/GBC Events (111)	Adjusted HR (95% CI)	Variable	*n* (%)	HGD/GBC Events (111)	Adjusted HR (95% CI)
WHtR quartiles				WHtR Chile BiLS categories			
Q1 (0.38–0.59)	1129 (25.0)	31 (27.9)	1.74 (0.99–3.06)	0.38–0.60	1218 (26.9)	33 (29.7)	1.79 (1.08–2.97)
Q2 (0.60–0.65)	1121 (24.8)	20 (18.0)	1.00 (ref)	0.61–0.68	1645 (36.4)	29 (26.1)	1.00 (ref)
Q3 (0.66–0.71)	1129 (25.0)	22 (19.8)	1.00 (0.54–1.83)	0.69–0.72	643 (14.2)	16 (14.4)	1.55 (0.87–2.75)
Q4 (0.72–1.22)	1144 (25.2)	38 (34.2)	1.48 (0.88–2.57)	0.73–0.77	564 (12.5)	23 (20.7)	1.73 (0.96–3.12)
				0.78–1.22	453 (10.0)	10 (9.0)	0.96 (0.48–1.94)
Global *p*	0.11			Global *p*	0.10		

Abbreviations: GBC, gallbladder cancer; HGD, high-grade dysplasia; WHtR, waist-to-height ratio; HR, hazard ratio; CI, confidence interval; BiLS, Chile Biliary Longitudinal Study. Footnote: The analytic sample was restricted to participants with available WHtR and covariate data (*n* = 4523). Of the 116 HGD/GBC cases identified during follow-up, 111 were included in the adjusted analyses; five cases were excluded because of missing covariate data. All frequencies are based on baseline measurements, whereas hazard ratios (HRs) were estimated from models with time-varying WHtR. Models were estimated using time-dependent Cox proportional hazards regression with age as the underlying timescale and were adjusted for parity (continuous), health coverage (FONASA), and self-reported Mapuche Amerindian ancestry. Panel A presents WHtR quartiles. Panel B presents Chile BiLS-specific WHtR categories, defined based on the shape of the restricted cubic spline fit for time-varying WHtR in the Chile BiLS cohort.

## Data Availability

The data relevant to the analyses and supporting the conclusions of this article will be available upon request for participants who consented to data sharing, excluding sensitive information such as Mapuche status. Requests for data access should be directed to Dr. Jill Koshiol (koshiolj@mail.nih.gov).

## Supplementary Materials

The following supporting information can be downloaded at https://www.mdpi.com/article/10.3390/cancers18203356/s1. Table S1: Sensitivity analyses of the association between spline-informed time-varying waist-to-height ratio (WHtR) categories and HGD/GBC after excluding events occurring early in follow-up, Chile BiLS, 2016–2025. Table S2: Adjusted time-dependent Cox models (age as the underlying timescale) for gallbladder cancer (GBC) and high-grade dysplasia (HGD) according to spline-informed BMI categories, women, Chile BiLS, 2016–2025. Table S3: Adjusted time-dependent Cox model (age as the underlying timescale) for gallbladder cancer (GBC) and high-grade dysplasia (HGD) according to spline-informed WHtR categories, women, Chile BiLS, 2016–2025. Table S4: Case-case analysis comparing high-grade dysplasia (HGD) with surgically detected gallbladder cancer (GBC) according to baseline waist-to-height ratio (WHtR), Chile BiLS, 2016–2025. Table S5. Case-case analysis comparing HGD/surgically detected GBC with clinically detected GBC according to baseline waist-to-height ratio (WHtR), Chile BiLS, 2016–2025. Figure S1: Individual and mean waist-to-height ratio (WHtR) trajectories over time before diagnosis of gallbladder cancer or high-grade dysplasia in Chilean women (2016–2025), for WHtR Categories 3 (0.69–0.72), 4 (0.73–0.77), and 5 (0.78–1.22), with 95% confidence intervals. Figure S2: Individual and mean waist-to-height ratio (WHtR) trajectories over time before cholecystectomy in Chilean women (2016–2025), for WHtR Categories 3 (0.69–0.72), 4 (0.73–0.77), and 5 (0.77–1.21), with 95% confidence intervals.

## References

[1] Gallbladder Cancer|Nature Reviews Disease Primers. https://www.nature.com/articles/s41572-022-00403-4.

[2] Filho A.M., Laversanne M., Ferlay J., Colombet M., Pineros M., Znaor A., Parkin D.M., Soerjomataram I., Bray F. (2025). The GLOBOCAN 2022 cancer estimates: Data sources, methods, and a snapshot of the cancer burden worldwide. Int. J. Cancer.

[3] Segundo Informe Registros Poblacionales de Cancer-Febrero2023|PDF|Cáncer|Cáncer de Pulmón. https://es.scribd.com/document/663656617/Segundo-Informe-Registros-Poblacionales-de-Cancer-Febrero2023.

[4] Lammert F., Gurusamy K., Ko C.W., Miquel J.-F., Méndez-Sánchez N., Portincasa P., van Erpecum K.J., van Laarhoven C.J., Wang D.Q.-H. (2016). Gallstones. Nat. Rev. Dis. Primer.

[5] Hsing A.W., Bai Y., Andreotti G., Rashid A., Deng J., Chen J., Goldstein A.M., Han T., Shen M., Fraumeni J.F. (2007). Family history of gallstones and the risk of biliary tract cancer and gallstones: A population-based study in Shanghai, China. Int. J. Cancer.

[6] Roa E.I., Muñoz N.S., Ibacache S.G., de Aretxabala U.X. (2009). Natural history of gallbladder cancer Analysis of biopsy specimens. Rev. Médica Chile.

[7] Roa J.C., Basturk O., Adsay V. (2021). Dysplasia and carcinoma of the gallbladder: Pathological evaluation, sampling, differential diagnosis and clinical implications. Histopathology.

[8] Zhou J., Yu W., Jiang G., Li H., Luo J., Li S., Xie L., Bai X., Xia J., Mao M. (2025). Risk of Gallstones Increases with Multiple Dimensions of Obesity Indexes: A Prospective Study Based on the UK Biobank. Obes. Facts.

[9] Zhang J., Chen L., Shen K., Zhang J., Zhu Y., Qiao Q., Chen L. (2023). Association between metabolically healthy overweight/obesity and gallstones in Chinese adults. Nutr. Metab..

[10] Maclure K.M., Hayes K.C., Colditz G.A., Stampfer M.J., Speizer F.E., Willett W.C. (1989). Weight, Diet, and the Risk of Symptomatic Gallstones in Middle-Aged Women. N. Engl. J. Med..

[11] Lee F., Moore J., Markouli M., Ghusn W. (2025). The Oncogenic Burden of Obesity: Mechanistic Links Between Adiposity and Gastrointestinal Cancers—A Comprehensive Narrative Review. Biomedicines.

[12] Ashwell M., Gibson S. (2016). Waist-to-height ratio as an indicator of ‘early health risk’: Simpler and more predictive than using a ‘matrix’ based on BMI and waist circumference. BMJ Open.

[13] Barberio A.M., Alareeki A., Viner B., Pader J., Vena J.E., Arora P., Friedenreich C.M., Brenner D.R. (2019). Central body fatness is a stronger predictor of cancer risk than overall body size. Nat. Commun..

[14] Li Z.M., Wu Z.X., Han B., Mao Y.-Q., Chen H.-L., Han S.-F., Xia J.-L., Wang L.-S. (2016). The association between BMI and gallbladder cancer risk: A meta-analysis. Oncotarget.

[15] Koshiol J., Van De Wyngard V., McGee E.E., Cook P., Pfeiffer R.M., Mardones N., Medina K., Olivo V., Pettit K., Jackson S.S. (2021). The Chile Biliary Longitudinal Study: A Gallstone Cohort. Am. J. Epidemiol..

[16] Rothman K.J., Greenland S., Lash T.L. (2008). Modern Epidemiology.

[17] Campbell P.T., Newton C.C., Kitahara C.M., Patel A.V., Hartge P., Koshiol J., McGlynn K.A., Adami H.-O., de González A.B., Freeman L.E.B. (2017). Body size indicators and risk of gallbladder cancer: Pooled analysis of individual-level data from 19 prospective cohort studies. Cancer Epidemiol. Biomark. Prev..

[18] Liu H., Zhang Y., Ai M., Wang J., Jin B., Teng Z., Wang Y., Li L. (2016). Body Mass Index Can Increase the Risk of Gallbladder Cancer: A Meta-Analysis of 14 Cohort Studies. Med. Sci. Monit. Basic Res..

[19] Jayedi A., Soltani S., Zargar M.S., Khan T.A., Shab-Bidar S. (2020). Central fatness and risk of all cause mortality: Systematic review and dose-response meta-analysis of 72 prospective cohort studies. BMJ.

[20] Sun Y., Liu B., Snetselaar L.G., Wallace R.B., Caan B.J., Rohan T.E., Neuhouser M.L., Shadyab A.H., Chlebowski R.T., Manson J.E. (2019). Association of Normal-Weight Central Obesity With All-Cause and Cause-Specific Mortality Among Postmenopausal Women. JAMA Netw. Open.

[21] Jackson S.S., Van Dyke A.L., Zhu B., Pfeiffer R.M., Petrick J.L., Adami H.-O., Albanes D., Andreotti G., Freeman L.E.B., de González A.B. (2019). Anthropometric Risk Factors for Cancers of the Biliary Tract in the Biliary Tract Cancers Pooling Project. Cancer Res..

[22] Ralston M.R., McCreath G., Lees Z.J., Salt I.P., Sim M.A., Watson M.J., Freeman D.J. (2025). Beyond body mass index: Exploring the role of visceral adipose tissue in intensive care unit outcomes. BJA Open.

[23] Xu F.Q., Xu Q.Y., Zhu Z.J., Jin L., Ye T., Du C., Gao Z., Huang X., Zhang Z., Jin L. (2024). Visceral and ectopic fat are more predictively associated with primary liver cancer than overall obesity from genetic sights: A Mendelian randomization study. Int. J. Cancer.

[24] Jovanović M., Kovačević S., Brkljačić J., Djordjevic A. (2023). Oxidative Stress Linking Obesity and Cancer: Is Obesity a ‘Radical Trigger’ to Cancer?. Int. J. Mol. Sci..

[25] Piovani D., Nikolopoulos G.K., Figlioli G., Tsantes A.G., Bonovas S. (2025). Global Burden of Gallbladder Cancer Attributable to Modifiable Risk Factors. Aliment Pharmacol. Ther..

[26] Watts E.L., Gonzalez-Feliciano A., Gunter M.J., Chatterjee N., Moore S.C. (2025). Adiposity and cancer: Systematic review and meta-analysis. medRxiv.

[27] Li L., Gan Y., Li W., Wu C., Lu Z. (2016). Overweight, obesity and the risk of gallbladder and extrahepatic bile duct cancers: A meta-analysis of observational studies. Obesity.

[28] Ponce C.B., Scherer D., Brinster R., Boekstegers F., Marcelain K., Gárate-Calderón V., Müller B., de Toro G., Retamales J., Barajas O. (2021). Gallstones, Body Mass Index, C-Reactive Protein, and Gallbladder Cancer: Mendelian Randomization Analysis of Chilean and European Genotype Data. Hepatology.

[29] Castle P.E., Wacholder S., Lorincz A.T., Scott D.R., Sherman M.E., Glass A.G., Rush B.B., Schussler J.E., Schiffman M. (2002). A Prospective Study of High-Grade Cervical Neoplasia Risk Among Human Papillomavirus-Infected Women. JNCI J. Natl. Cancer Inst..

[30] Dolcini J., Chiavarini M., Firmani G., Ponzio E., D’Errico M.M., Barbadoro P. (2024). Consumption of Bottled Water and Chronic Diseases: A Nationwide Cross-Sectional Study. Int. J. Environ. Res. Public Health.

