# Supplementary material for: Obesity and Risk of High-Grade Dysplasia and Gallbladder Cancer Among Women with Gallstones in the Chile Biliary Longitudinal Study
Source: Cancers (Basel). 2026 Oct 16;18(20):3356. doi: 10.3390/cancers18203356 (PMC13702334; doi:10.3390/cancers18203356)
Supplement: Supplementary file 1 [file cancers-18-03356-s001.zip › cancers-4591059-supplementary.pdf]

SUPPLEMENTAL TABLES

Supplementary Table S1. Sensitivity analyses of the association between spline-informed time-varying waist-to-height ratio (WHtR) categories and HGD/GBC after excluding events occurring early in follow-up, Chile BiLS, 2016–2025.

**Time-varying WHtR, Chile BiLS categories**	**6-month lag aHR (95% CI)**	**1-year lag aHR (95% CI)**	**2-year lag aHR (95% CI)**
0.38–0.60	1.71 (1.00–2.91)	1.59 (0.92–2.76)	1.89 (1.00–3.59)
0.61–0.68	1.00 (ref)	1.00 (ref)	1.00 (ref)
0.69–0.72	1.57 (0.87–2.85)	1.46 (0.78–2.70)	1.88 (0.94–3.77)
0.73–0.77	1.55 (0.83–2.90)	1.40 (0.73–2.71)	1.76 (0.83–3.70)
0.78–1.22	1.01 (0.50–2.06)	1.05 (0.51–2.14)	1.10 (0.47–2.57)
HGD/GBC events, *n*	101	93	73
p-overall	0.23	0.47	0.22

Abbreviations: GBC, gallbladder cancer; HGD, high-grade dysplasia; WHtR, waist-to-height ratio; HR, hazard ratio; CI, confidence interval; BiLS, Chile Biliary Longitudinal Study.

Footnote: Hazard ratios (HRs) and 95% confidence intervals (CIs) were estimated using time-dependent Cox proportional hazards regression with age as the underlying timescale. Models were adjusted for parity (continuous), health coverage (FONASA), and self-reported Mapuche Amerindian ancestry. Sensitivity analyses using the spline-informed WHtR categories, excluded HGD/GBC events occurring within the first 6 months, 1 year, and 2 years of follow-up, respectively. *p*-overall values correspond to Type 3 Wald tests for the overall association of time-varying WHtR categories with HGD/GBC risk.

Supplementary Table S2. Adjusted time-dependent Cox models (age as the underlying timescale) for gallbladder cancer (GBC) and high-grade dysplasia (HGD) according to spline-informed BMI categories, women, Chile BiLS, 2016-2025.

	**Clinical GBC cases[a] (*n*=1774, 21 events)**		**Cholecystectomy GBC cases[b] (*n*=2743, 45 events)**		**Cholecystectomy HGD[c] cases (*n*=2743, 45 events)**	
**Variable**	**(*n*) %**	**HR (95% CI)**	**(*n*) %**	**HR (95% CI)**	**(*n*) %**	**HR (95% CI)**
BMI Chile BiLS Categories spline informed						
15.9–28.0	6 (28.6)	1.03 (0.38–2.81)	14 (31.1)	1.86 (0.89–3.87)	13 (28.9)	1.68 (0.78–3.60)
28.1–34.0	10 (47.6)	1.00 (ref)	16 (35.6)	1.00 (ref)	14 (31.1)	1.00 (ref)
34.1–36.0	2 (9.5)	0.35 (0.05–2.72)	7 (15.6)	1.12 (0.37–3.41)	3 (6.7)	0.77 (0.22–2.68)
36.1–40.0	2 (9.5)	0.23 (0.03–1.78)	5 (11.1)	1.60 (0.67–3.83)	10 (22.2)	1.42 (0.57–3.53)
40.1–71.2	1 (4.8)	0.40 (0.09–1.83)	3 (6.7)	1.41 (0.46–4.30)	5 (11.1)	2.38 (0.99–5.70)

Abbreviations: BMI, body mass index; GBC, gallbladder cancer; HGD, high-grade dysplasia; HR, hazard ratio; CI, confidence interval; BiLS, Chile Biliary Longitudinal Study.

Footnote: All frequencies were calculated at baseline, whereas HRs were estimated from time-varying analysis. Models were estimated using time-dependent Cox proportional hazards regression with age as the underlying timescale.

BMI Chile BiLS–specific categories were defined based on the shape of restricted cubic spline fits for time-varying BMI in the Chile BiLS cohort. All models were adjusted for parity (continuous), health coverage (FONASA), and self-reported Mapuche Amerindian ancestry.

a Clinical GBC cases are gallbladder cancer diagnoses identified clinically through cancer registry or medical records and not associated with the cholecystectomy specimen.

b Surgical GBC cases are gallbladder cancer diagnoses made histologically in the cholecystectomy specimen.

c Surgical HGD cases are high-grade dysplasia lesions identified histologically in the cholecystectomy specimen.

Supplementary Table S3. Adjusted time-dependent Cox model (age as the underlying timescale) for gallbladder cancer (GBC) and high-grade dysplasia (HGD) according to spline-informed WHtR categories , women, Chile BiLS, 2016-2025.

	**Clinical GBC cases[a] (*n*=1777, 21 events)**		**Cholecystectomy GBC cases[b] (*n*=2746, 45 events)**		**Cholecystectomy HGD[c] cases (*n*=2746, 45 events)**		**Cholecystectomy HGD/GBC cases (*n*=2746, 90 events)**	
**Variable**	**(*n*) %**	**HR (95% CI)**	**(*n*) %**	**HR (95% CI)**	**(*n*) %**	**HR (95% CI)**	**(*n*) %**	**HR (95% CI)**
**WHtR Chile BiLS Categories (spline-informed)**								
0.38–0.60	5 (23.8)	1.61 (0.52–5.00)	12 (26.7)	1.40 (0.62–3.13)	16 (35.6)	2.30 (1.02–5.17)	28 (31.1)	1.77 (1.00–3.12)
0.61–0.68	7 (33.3)	1.00 (ref)	12 (26.7)	1.00 (ref)	10 (22.2)	1.00 (ref)	22 (24.4)	1.00 (ref)
0.69–0.72	3 (14.3)	1.56 (0.47–5.16)	9 (20.0)	1.51 (0.59–3.87)	4 (8.9)	1.75 (0.69–4.47)	13 (14.4)	1.62 (0.84–3.14)
0.73–0.77	4 (19.0)	0.69 (0.14–3.45)	7 (15.6)	2.10 (0.88–5.03)	12 (26.7)	2.10 (0.82–5.36)	19 (21.1)	2.10 (1.11–3.96)
0.78–1.22	2 (9.5)	0.55 (0.11–2.76)	5 (11.1)	1.77 (0.62–5.06)	3 (6.7)	1.47 (0.46–4.72)	8 (9.0)	1.60 (0.73–3.49)

Abbreviations: GBC, gallbladder cancer; HGD, high-grade dysplasia; WHtR, waist-to-height ratio; HR, hazard ratio; CI, confidence interval; BiLS, Chile Biliary Longitudinal Study.

Footnote: Figure 1 presents all HGD/GBC cases identified during follow-up, whereas the outcome-specific models presented in this table were restricted to participants with complete exposure and covariate data. Accordingly, the adjusted analyses included 45 of 49 cholecystectomy-detected GBC cases, 21 of 22 clinically detected GBC cases, and all 45 HGD cases (Total *n*=111). The cholecystectomy HGD/GBC analysis combined the 45 HGD and 45 cholecystectomy-detected GBC cases (90 events) and was restricted to participants with cholecystectomy-detected outcomes.

All frequencies were calculated at baseline, whereas HRs were estimated from time-varying analysis. Models were estimated using time-dependent Cox proportional hazards regression with age as the underlying timescale. WHtR Chile BiLS–specific categories were defined based on the shape of restricted cubic spline fits for time-varying WHtR in the Chile BiLS cohort. Models were adjusted for parity (continuous), health coverage (FONASA), and self-reported Mapuche Amerindian ancestry.

a Clinical GBC cases are gallbladder cancer diagnoses identified clinically through cancer registry or medical records and not associated with the cholecystectomy specimen.

b Cholecystectomy GBC cases are gallbladder cancer diagnoses made histologically in the cholecystectomy specimen.

c Cholecystectomy HGD cases are high-grade dysplasia lesions identified histologically in the cholecystectomy specimen.

Supplementary Table S4. Case-case analysis comparing high-grade dysplasia (HGD) with surgically detected gallbladder cancer (GBC) according to baseline waist-to-height ratio (WHtR), Chile BiLS, 2016–2025.

**Baseline WHtR category**	**HGD, *n* (%)**	**Surgically detected GBC, *n* (%)**	**Adjusted OR (95% CI)**
0.38–0.60	16 (35.6)	12 (26.7)	2.16 (0.58–8.01)
0.61–0.68	10 (22.2)	12 (26.7)	1.00 (ref)
0.69–0.72	4 (8.9)	9 (20.0)	0.46 (0.09–2.31)
0.73–0.77	12 (26.7)	7 (15.6)	2.29 (0.58–9.04)
0.78–1.22	3 (6.7)	5 (11.1)	0.88 (0.14–5.60)
p-overall			0.23

Abbreviations: GBC, gallbladder cancer; HGD, high-grade dysplasia; WHtR, waist-to-height ratio; OR, odds ratio; CI, confidence interval; FONASA, Fondo Nacional de Salud; BiLS, Chile Biliary Longitudinal Study.

Footnote: Frequencies were calculated using baseline WHtR categories. ORs and 95% CIs were estimated using multivariable logistic regression comparing HGD with surgically detected GBC among outcomes identified through cholecystectomy. The adjusted analysis included 90 participants (45 HGD and 45 surgically detected GBC cases). Models were adjusted for age at enrollment, parity, health coverage (FONASA), and self-reported Mapuche Amerindian ancestry. *p*-overall corresponds to the Type 3 Wald test for the overall association of baseline WHtR category with case type.

Supplementary Table S5. Case-case analysis comparing HGD/surgically detected GBC with clinically detected GBC according to baseline waist-to-height ratio (WHtR), Chile BiLS, 2016–2025.

**Baseline WHtR category**	**HGD/surgically detected GBC, *n* (%)**	**Clinically detected GBC, *n* (%)**	**Adjusted OR (95% CI)**
0.38–0.60	28 (31.1)	5 (23.8)	1.28 (0.29–5.60)
0.61–0.68	22 (24.4)	7 (33.3)	1.00 (ref)
0.69–0.72	13 (14.4)	3 (14.3)	1.01 (0.19–5.36)
0.73–0.77	19 (21.1)	4 (19.0)	1.28 (0.30–5.49)
0.78–1.22	8 (9.0)	2 (9.5)	1.45 (0.21–10.00)
p-overall			0.99

Abbreviations: GBC, gallbladder cancer; HGD, high-grade dysplasia; WHtR, waist-to-height ratio; OR, odds ratio; CI, confidence interval; FONASA, Fondo Nacional de Salud; BiLS, Chile Biliary Longitudinal Study.

Footnote: Frequencies were calculated using baseline WHtR categories. ORs and 95% CIs were estimated using multivariable logistic regression comparing HGD/surgically detected GBC with clinically detected GBC. The adjusted analysis included 111 participants (90 HGD/surgically detected GBC cases and 21 clinically detected GBC cases). Models were adjusted for age at enrollment, parity, health coverage (FONASA), and self-reported Mapuche Amerindian ancestry. *p*-overall corresponds to the Type 3 Wald test for the overall association of baseline WHtR category with mode of outcome ascertainment.

Supplemental figures

Figure S1. Individual and mean waist-to-height ratio (WHtR) trajectories over time before diagnosis of gallbladder cancer or high-grade dysplasia in Chilean women (2016-2025), for WHtR Categories 3 (0.69–0.72), 4 (0.73–0.77), and 5 (0.78–1.22), with 95% confidence intervals.

1) **WHtR Category 3: 0.69-0.72**

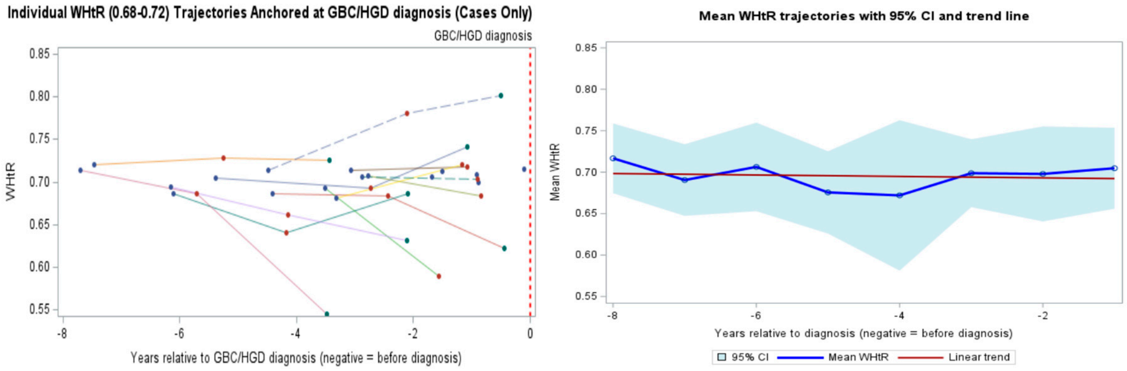

2) **WHtR Category 4: 0.73-0.77**

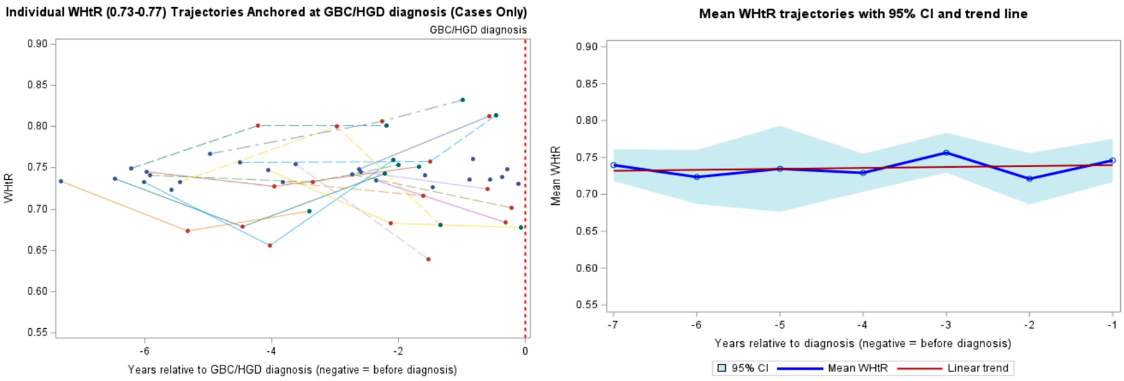

3) **WHtR Category 5: 0.78-1.22**

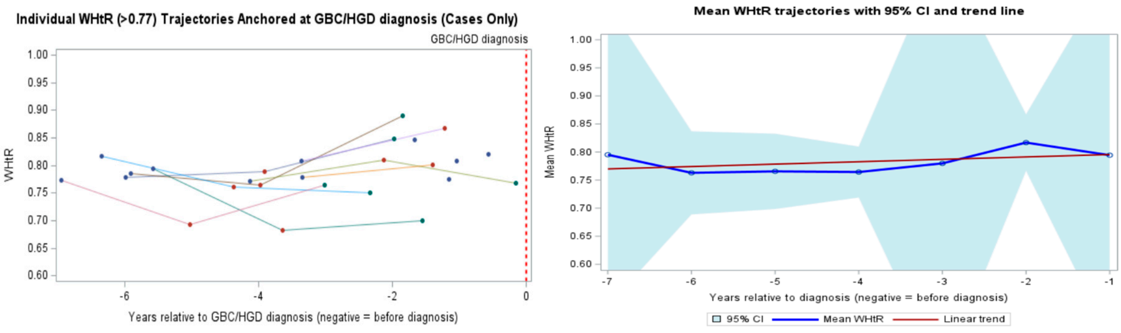

Footnote: Lines represent fitted linear trajectories of WHtR over time among cases (n=116; 235 visits). Overall slope p=0.054; estimated annual change in WHtR by baseline category: Cat3 (0.69–0.72) +0.0018/year (p=0.23), Cat4 (0.73–0.77) +0.0007/year (p=0.79), Cat5 (0.78–1.22) +0.0058/year (p=0.18).

Alt text: Figure S1 showing individual and mean waist-to-height ratio (WHtR) trajectories over time before diagnosis of gallbladder cancer or high-grade dysplasia in Chilean women (2016–2025). The figure has three horizontal panels corresponding to WHtR Categories 3 (0.69–0.72), 4 (0.73–0.77), and 5 (0.78–1.22). In each panel, the left plot displays colored lines representing individual WHtR measurements for cases during the years preceding diagnosis, with the x-axis indicating time in years relative to diagnosis (negative values occurring earlier, zero at diagnosis) and the y-axis showing WHtR values; a vertical dashed red line marks the time of diagnosis. In each panel, the right plot shows a solid blue line for the mean WHtR trajectory over the same time window, surrounded by a light blue shaded band depicting the 95% confidence interval and a darker line indicating the fitted linear trend, allowing visual comparison of average WHtR levels, trends, and uncertainty across WHtR categories in the pre-diagnostic period.

Figure S2. Individual and mean waist-to-height ratio (WHtR) trajectories over time before cholecystectomy in Chilean women (2016-2025), for WHtR Categories 3 (0.69–0.72), 4 (0.73–0.77), and 5 (0.77–1.21), with 95% confidence intervals.

1) WHtR Category 3: 0.69-0.72

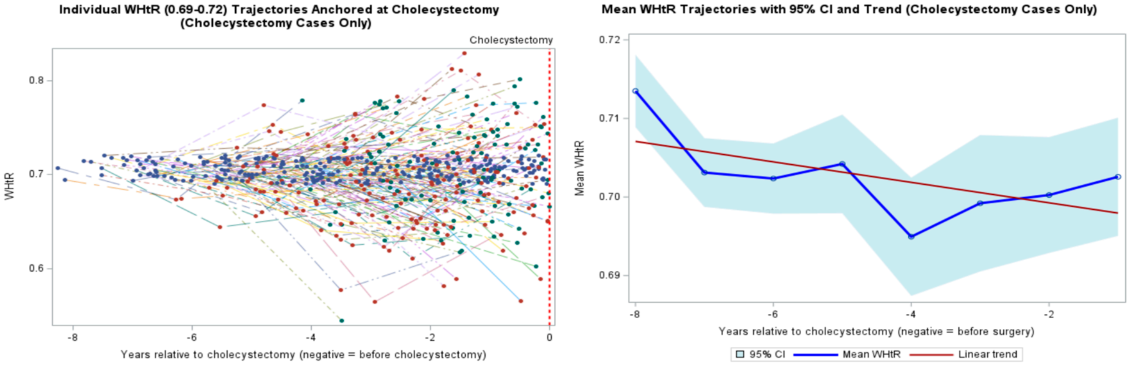

2) WHtR Category 4: 0.73-0.77

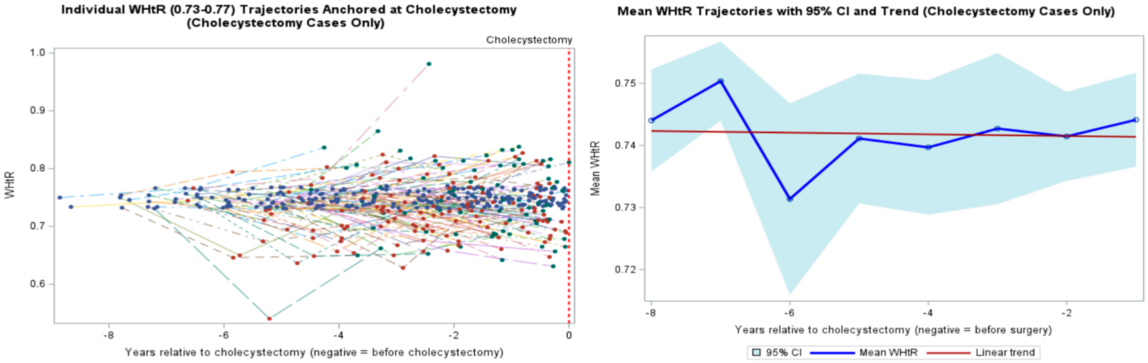

3) WHtR Category 5: 0.78-1.22

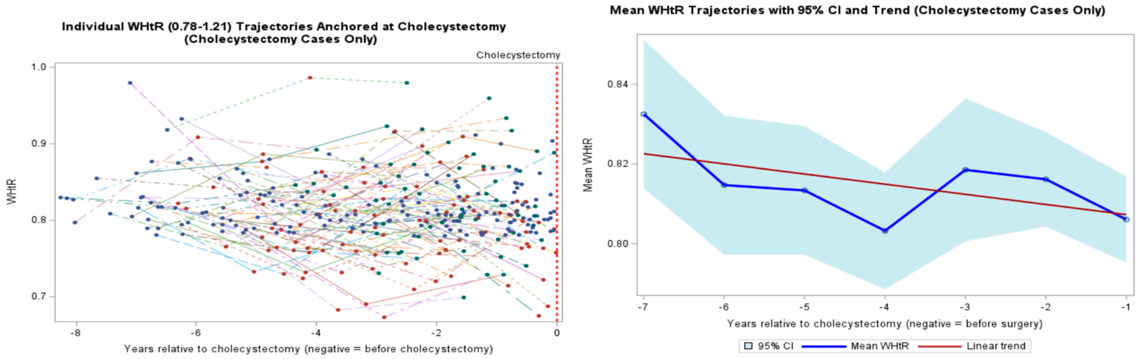

Footnote: Among 2,828 women (5,333 observations) anchored at cholecystectomy, estimated annual change in WHtR was: Cat3 (0.69–0.72) +0.00020/year (p=0.49), Cat4 (0.73–0.77) -0.00050/year (p=0.49), Cat5 (0.78-1.22) -0.00181/year (p=0.03).

Alt text: Figure S2 showing individual and mean waist-to-height ratio (WHtR) trajectories over time before cholecystectomy in Chilean women (2016–2025). The figure has three horizontal panels, corresponding to WHtR categories 3 (0.69–0.72), 4 (0.73–0.77), and 5 (0.78–1.22). In each panel, the left plot displays multiple colored lines representing individual WHtR measurements for women during the years preceding surgery, with the x-axis showing time in years before cholecystectomy (values closer to zero indicating proximity to surgery) and the y-axis showing WHtR values. In each panel, the right plot shows a solid line for the mean WHtR trajectory over the same time scale, surrounded by a shaded band indicating the 95% confidence interval around the mean, allowing visual comparison of average WHtR levels and uncertainty across categories in the years leading up to surgery.
